# Laboratory and experimental hut evaluation of a long-lasting insecticide treated blanket for protection against mosquitoes

**DOI:** 10.1186/1756-3305-7-129

**Published:** 2014-03-28

**Authors:** Jovin Kitau, Richard Oxborough, Angela Kaye, Vanessa Chen-Hussey, Evelyn Isaacs, Johnson Matowo, Harparkash Kaur, Stephen M Magesa, Franklin Mosha, Mark Rowland, James Logan

**Affiliations:** 1 Department of Entomology and Parasitology, Kilimanjaro Christian Medical University College (KCMUCo), Moshi, Tanzania; 2 Pan-African Malaria Vector Research Consortium, (PAMVERC), Moshi, Tanzania; 3 Department of Disease Control, London School of Hygiene and Tropical Medicine (LSHTM), London, UK; 4 Africa University Development Office, Nashville, TN 37203-0007, USA; 5 RTI International, Global Health Division, Nairobi, Kenya

**Keywords:** Insecticide treated blankets, Pyrethroids, *Anopheles arabiensis*, Vector control, Experimental hut, Tanzania

## Abstract

**Background:**

Long-lasting insecticide treated blankets (LLIBs) may provide additional protection against malaria where use of long lasting insecticidal nets (LLIN) is low or impractical such as in disaster or emergency situations.

**Methods:**

Initial efficacy testing of a new candidate LLIB was carried out at LSHTM and KCMUCo, before and after washing, in cone and ball bioassays and arm-in-cage tests against pyrethroid susceptible *Anopheles gambiae*. A small scale field trial was conducted using veranda-trap experimental huts in northern Tanzania against wild *An. arabiensis* and *Culex quinquefasciatus* mosquitoes. Treatments included unwashed and 5 times washed permethrin treated LLIB and blankets hand-treated with permethrin (ITB), untreated blankets, and a holed unwashed Olyset net.

**Results:**

Cone test mortality was 75% for LLIB when unwashed, but decreased to 32% after 5 washes and <10% after 10 washes. In arm-in-cage tests protection against biting was 100% for LLIBs regardless of the number of washes while reduction in landings was 79% when unwashed, 75% after 5 washes, but declined to 41% after 10 and 33% after 20 washes. In ball bioassays using pyrethroid resistant *An. arabiensis*, mortality was low in all treatments (<35%) and there was no significant difference in mortality between Olyset net, LLIB or ITB (p > 0.05). Percentage mortality of *An. arabiensis* in huts with LLIB unwashed (26%) was not statistically different to Olyset net (31%, p = 0.5). The 5 times washed LLIB reduced blood-feeding by 49% which was equivalent to Olyset net (p > 0.086). There was no significant difference in percentage blood-feeding between LLIB and ITB unwashed or 5 times washed (p = 0.147 and p = 0.346 respectively). The 5 times washed LLIB reduced blood-feeding of *Culex quinquefasciatus* by 40%, although the Olyset provided the greatest protection with 85% inhibition. ELISA analysis of a sub-sample of blood fed mosquitoes showed that not all had fed on humans in the huts, therefore blood-feeding inhibition may have been underestimated.

**Conclusions:**

This trial demonstrated the potential of LLIBs to provide substantial personal protection even against pyrethroid resistant mosquitoes. LLIBs may prove particularly useful where LLINs are unsuitable or net usage is low.

## Background

Insecticide treated mosquito nets (ITNs), Long Lasting Insecticidal Nets (LLINs) and indoor residual spraying (IRS) are the most advocated malaria vector control tools. They have been proven to be effective across a range of transmission settings in reducing malaria transmission and disease burden [1,2]. In emergency situations such as civil or political conflicts and natural disasters where populations are displaced, or in nomadic groups, LLINs and IRS become impractical [3]. Refugee camps often lack appropriate substrates such as walls and ceilings for spraying with insecticides, and nets cannot be easily hung in refugee tents [3,4].

The United Nations High Commissioner for refugees (UNHCR) estimated that there were 2,774,500 refugees in Africa at the end of 2012, with the majority present in Eastern and Horn Regions of Africa. The response to humanitarian emergencies is to provide untreated blankets/sheets/tents to displaced populations for warmth and shelter. Factory treatment of these materials with insecticides could results in large-scale, long-term protection against disease vectors at low additional cost [3,5]. Despite increased displacement in Africa [6] there is still limited distribution of treated blankets and clothing [7].

Pyrethroid insecticides have low mammalian toxicity and have been used safely on clothing and other materials that contact the skin for several decades [8,9]. Pyrethroid treated fabrics like tents, top-sheets, blankets, tarpaulins, hammocks and chaddars are potential tools for control of mosquitoes and other disease vectors [4,5,10-15]. Rowland *et al.*[10] demonstrated that permethrin impregnated sheets and chaddars used in an Afghan refugee camp reduced mosquito blood-feeding success on humans by 70% and substantially reduced malaria episodes for both *Plasmodium falciparum* and *P. vivax*. The use of permethrin-treated clothes and bedding materials (blankets and sheets) in a Dadaab refugee camp in North East Kenya reduced both indoor mosquito population densities and malaria infection rates (OR = 0.31) [16].

While the emphasis has been on emergencies there is increasing evidence that insecticide treated blankets and sheets may prove equally useful in peacetime communities as a supplement to LLINs and IRS. Even at high levels of ownership, regular usage of LLINs by households cannot be guaranteed. In rural Tanzania in 2011 following the Universal Coverage Campaign of LLINs (UCC) the number of nets owned nearly doubled but usage only increased from 41% to 56% [17]. In Kenya, Atieli *et al.*[18] recorded seasonal variation in ITN usage, with lowest usage (49%) in the dry season compared to 62% usage during the rainy season. Pyrethroid treated sheets and lightweight blankets are more likely to be used year-round, thereby potentially overcoming the limitation of bed net usage. Insecticide treated blankets may also provide additional protection when used in a household in addition to using ITNs or IRS.

The aim of this study was to determine the efficacy of a Long-lasting permethrin treated blanket (LLIB) relative to a hand-dipped permethrin treated blanket (ITB) and Olyset LLIN against laboratory-reared and wild, free-flying, *Anopheles arabiensis* and *Culex quinquefasciatus* mosquitoes in Tanzania.

## Methods

### Study sites

Initial repellency and insecticidal testing, plus chemical analysis of LLIBs washed 0, 5, 10, 15 and 20 times was conducted by the Arthropod Control Product Test Centre (*arctec)* at the London School of Hygiene and Tropical Medicine (LSHTM), UK (Table 1). Subsequently, based on the LSHTM data, unwashed and 5 times washed treatments were assayed at the Kilimanjaro Christian Medical University College (KCMUCo) and an experimental hut trial was carried out in Lower Moshi (3°22′S, 37°19′E; altitude 800 m). Lower Moshi is an intensively farmed, irrigated rice-growing area where *An. arabiensis* and *Cx. quinquefasciatus* are the predominant species. *Cx. quinquefasciatus* were moderately resistant to deltamethrin and permethrin recorded from mortalities (51.5% and 68.0%, respectively) in WHO susceptibility tests [19]. *Anopheles arabiensis* from lower Moshi have been found to be susceptible to organophosphates and carbamates [20]. *Anopheles arabiensis* were mostly susceptible to pyrethroid insecticides in 2006 and 2010 [20,21], but more recent studies have demonstrated an increase in the resistance frequency due to raised levels of mixed function oxidases and non-specific esterases (Matowo unpublished data).

**Table 1:** Location and type of testing carried out on Long-lasting insecticidal blankets.

**Location**	**Materials tested**	**Test type**	**Mosquito species**
LSHTM, UK	Blankets: 0, 5, 10, 15 and 20 washes	Cone bioassay	*An. gambiae* (Kisumu)
		Arm-in-cage bioassays	*An. gambiae* (Kisumu)
		HPLC	N/A
KCMUCo, Tanzania	Blankets : 0 and 5 washes Olyset net	Ball bioassays	*An. gambiae* (Kisumu)
			*An. arabiensis* (F1)
		Arm-in cage bioassay	*An. gambiae* (Kisumu)
		Experimental hut trial	*An. arabiensis*
			*Cx. quinquefasciatus*

### Laboratory studies

#### Insecticide treated blankets and untreated controls

The LLIB tested in laboratory and semi-field studies is a 1.7 × 2.4 m blanket (Berkley Medical Resources Inc., Pennsylvania, USA) impregnated using two binders with permethrin at a rate of 1130 mg/m^2^. Untreated blankets of the same material with no binder were used as negative controls. To compare the LLIBs with a home treatment method, a set of untreated blankets were hand-dipped in Tanzania, these are referred to as insecticide treated blankets (ITBs). The untreated blankets were strongly hydrophobic and initially would not easily absorb insecticide solution during dipping. Therefore, untreated blankets were first washed with soap and rinsed with water before treating with insecticide. Blankets were individually treated with a target dosage of 0.5 g/m^2^ using 4.16 ml of 50% Permethrin EC (Sumitomo Chemical Co. Ltd) mixed in 1130 millilitres of water; an amount sufficient to saturate the blanket without dripping. Blankets were kneaded until fully saturated. Dipped blankets were individually dried horizontally on plastic sheets in shade until fully dried and stored at room temperature. Unwashed Olyset nets (1000 mg/m^2^, Sumitomo Chemical Co. Ltd) were used as a positive control. The Olyset net was deliberately holed with six holes (4 cm × 4 cm) two on each long side and one on each short side to simulate a torn net.

#### Washing procedure

The washing procedures used were based on those described by the World Health Organization (WHO) for bioassay insecticide evaluations (WHO, 2005). For tests at LSHTM, test materials were cut into twenty 25 × 25 cm squares and introduced into a 1 L beaker containing 0.5 L of deionised water with 2 g/L of soap (savon de Marseille, pH 10–11). These were placed in a water bath at 30°C and shaken at 155 movements per minute for ten minutes. Samples were then rinsed twice with deionised water under the same conditions. The materials were dried at room temperature before repeating the process. The samples were washed 5, 10, 15 or 20 times, and were stored wrapped in aluminium foil at 30°C. In Tanzania, the procedure was slightly modified to more accurately reflect hand-washing practices. Whole blankets were washed for 10 minutes in aluminium vessels containing 15 L of tap water with 30 g of soap. Using a paddle, blankets were agitated at approximately 20 rotations per minute, both clockwise and anticlockwise. Each blanket was rinsed twice in a vessel with 15 L of clean water using the same number of agitations. After rinsing, blankets were dried horizontally on a plastic sheet in shade. Blankets were stored for 2 days between washes at ambient temperature of 20-25°C.

#### Mosquito strains

*Anopheles gambiae* s.s. Kisumu is a pyrethroid susceptible insectary strain originally from Kisumu, Kenya. Colonies reared in both London and Tanzania were used during testing. *Anopheles arabiensis* Moshi are the F1 offspring from wild *An. arabiensis* collected in Moshi, Tanzania and are partially resistant to pyrethroids. Mosquito rearing and insecticide testing conditions were held at 25 ± 2°C and 70 ± 10%.

#### Contact bioassays

Initial WHO cone bioassays were carried out at LSHTM (London, UK) to test the efficacy of treated and untreated blankets washed 0, 5, 10, 15 and 20 times. Ten squares, 7 × 7 cm, were cut from each of the untreated and treated blankets. Each piece of material was covered with a WHO bioassay cone, and secured in place with elastic bands to a clean white tile. Ten female *Anopheles gambiae* s.s. Kisumu (3–4 days old) were introduced to the cone and left for 3 minutes. The mosquitoes were then removed and placed in a recovery cup with access to 10% glucose solution. Mosquitoes were placed in a humidity chamber and knockdown recorded at 10 minutes post exposure and mortality after 24 hours.

Ball bioassays of blankets were conducted at KCMUCo (Tanzania). This is a WHO recommended method for testing contact efficacy of LLINs and insecticide-treated materials and ensures contact with the material for the duration of the exposure; whereas in cone tests with irritant insecticides the mosquito can avoid contact by resting on the plastic cone. Ball bioassays were performed on blankets that were later used in experimental hut trials. Wire ball frames were attached to blankets at random positions and ten unfed female mosquitoes were inserted into the ball frames and exposed for 3 minutes. The strains tested were *An. gambiae* s.s. Kisumu (pyrethroid susceptible) and *An. arabiensis* Moshi F1 offspring from mosquitoes collected in an area close to experimental hut sites (partially resistant to pyrethroids). To recover the mosquitoes after 3 minutes exposure, the ball was unwrapped inside a 30 × 30 × 30 cm cage. Mosquitoes were placed in a holding area with controlled temperature (22-27°C) and humidity (65-85%) and knockdown was recorded at 1 hour post exposure and mortality after 24 hours. Four replicates were done for each test material.

#### Arm-in-cage test for repellency

At LSHTM (UK) an arm-in-cage experiment was carried out on LLIBs and untreated blankets washed 0, 5, 10, 15 and 20 times. For these experiments thirty female *An. gambiae* Kisumu mosquitoes were introduced to a cage 30 × 30 × 30 cm. Readiness to bite was assessed before testing by placing a bare arm into the cage for up to 30 seconds. At least 10 mosquitoes landing on the arm were required to continue with the tests. If fewer than 10 mosquitoes landed, then the mosquitoes were discarded, new mosquitoes used and biting readiness re-assessed. Material sections were secured in place over the volunteer’s arm using tape. The control arm was then placed in the cage for 90 seconds and the number of mosquitoes landing was recorded. The treatment arm was then placed in the cage for 90 seconds and the number of mosquitoes landing was recorded. Bites were then assessed by examining the arms after 2 minutes. Three replicates were carried out for each treatment.

At KCMUCo (Tanzania), the same treatments used later in the experimental hut trial were tested in arm-in-cage tests to determine levels of repellency following a similar protocol as at LSHTM, except with exposure times of 5 minutes, and biting was not recorded. Three replicates were carried out for each treatment, with each replicate using a different volunteer.

### HPLC analysis of washed materials

At LSHTM (UK) samples of the LLIBs washed 0, 5, 10, 15, 20 times and tested in cone and arm-in-cage bioassays were tested by high performance liquid chromatography (HPLC) with photodiode array to assess the quantity of pyrethroid present on the material. Four 2.5 × 2.5 cm squares were cut from an LLIB and an untreated blanket. Each sample was extracted into 1 ml of acetonitrile and sonicated for 5 minutes before being transferred into the HPLC vials. HPLC analyses were carried out using a Dionex Ultimate 3000 range of equipment and software (Camberly, Surrey, UK). Samples were separated on an Acclaim^R^ C_18_ 120 Å (250 × 4.6 mm, Dionex, UK) column eluting with water/acetonitrile (90:10%; v/v) at a flow rate of 2 ml/min and passed through the photodiode array detector (PDA-100, Dionex) set at 275 nm. The authenticity of the detected peaks was determined by comparison of retention time, spectral extraction at 275 nm and spiking the sample with commercially available standard of the insecticide. A calibration curve of insecticide was generated by Chromeleon (Dionex software), using known amounts of the standard (0–0.4 μg/ml) in acetonitrile injected onto the column. From this curve the amount of insecticide in the matrix was calculated. Approximate doses of insecticide per m^2^ were calculated from the quantities detected in each 6.25 cm^2^ sample. The mean of the four determinations was then used to calculate the total insecticide on the material.

### Experimental hut study

#### Experimental huts

The experimental hut study was done in Lower Moshi. A suite of 7 experimental huts built to a traditional East African design as described in the WHOPES, “Guidelines for testing mosquito adulticides” [22-24] were used for the trial. Huts are built with brick walls plastered with mud on the inside, a wooden ceiling, a metal sheet covered roof, open eaves with window traps and verandah traps on each side. Minor modifications were made by installing wooden eave baffles on two sides (East and West) to still allow entry but prevent egress of mosquitoes that entered the hut. The other two eave sides were left open (un-baffled) so mosquitoes could exit and subsequently be collected in screened verandahs. The trial was done for seven weeks (49 collection nights) from July-September 2012. Seven treatments (six blankets and one LLIN) were tested in the huts: (i) unwashed untreated blanket, (ii) untreated blanket washed 5 times, (iii) unwashed permethrin factory treated (LLIB) blanket, (iv) permethrin factory treated (LLIB) blanket washed 5 times, (v) unwashed conventionally treated Permethrin blanket (ITB), (vi) conventionally treated Permethrin blanket (ITB) washed 5 times, (vii) unwashed Olyset net.

#### Hut procedure

Seven volunteers were recruited for the study and each slept individually in one of the huts every test night from 19:00–6:30 Hrs and were instructed to cover themselves with the blanket treatments or sleep under the net (Olyset). Each morning trained researchers collected mosquitoes from each hut using mouth-aspirators between 6:30–08:00 Hrs. Dead and alive mosquitoes were collected from the 2 screened verandas, bedroom, two window traps and inside the holed Olyset net. White plastic lining was laid on the floor to make dead mosquitoes more easily visible.

Live collected mosquitoes were kept in paper cups, provided with 10% glucose solution and kept in the field laboratory to score delayed mortality after 24 Hrs. Sleepers were alternated between huts on successive nights to reduce any bias due to differences in individual attractiveness to mosquitoes. Every 7 days the treatments were alternated between huts according to a Latin square design, leaving 1 day for aeration between treatment rotations. Two samples per treatment arm were used and exchanged nightly to capture any variation in sample preparation. Collected mosquitoes were identified and grouped as *Anopheles gambiae* or *Culex quinquefasciatus* based on their morphological characteristics [25,26] and recorded as unfed, blood-fed, semi-gravid, or gravid. *Anopheles gambiae* were assumed to be *An. arabiensis* based on previous PCR identification [21,27]. *Cx. quinquefasciatus* has been the predominant species of this genera found in the study area [28,29]. A small number were collected and recorded as other *Culex* species but not included in the analysis. All blood-fed mosquitoes collected on day 7 of each treatment rotation had their abdomens crushed on filter papers on the morning of collection. These mosquitoes were only used for blood-meal analysis and were not used as a measure of mortality or blood-feeding inhibition. Filter papers were stored in small plastic bags in a freezer for later identification of blood meal sources by enzyme-linked immunosorbent assay, ELISA.

The primary outcomes were:

– Blood feeding inhibition (the reduction in blood feeding in treatment huts relative to the control hut);

– Mortality (the proportion of mosquitoes killed out of total number collected).

A secondary outcome was:

– Induced exophily (the proportion of mosquitoes that were collected from exit traps and verandahs in treatment huts relative to control huts);

#### Blanket usage

**Figure 1:**
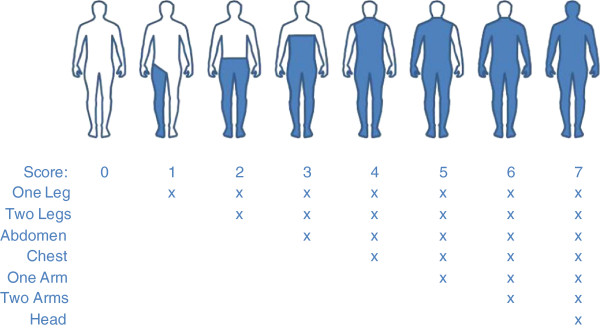
Body cover score system for sleepers using blankets.

Twice every test night a research scientist recorded how well sleepers covered themselves with the blankets by observing through a window at 22:00 and 05:00 Hrs. The records were filled in as body cover scores. For this the body was divided into seven general parts (left leg, right leg, left arm, right arm, abdomen, chest, head) each carrying one score (Figure 1); score of 7 (100%) meant a total body cover and 0 (0%) for no body part covered.

#### Blood meal analysis

Blood meal analysis of a sub-sample of fed mosquitoes was done by direct ELISA, using a methodology based on Beier *et al.*[30] to determine which host the mosquitoes had fed on. Only samples collected on the 7^th^ day of each rotation and immediately smeared on filter papers were analysed. Mosquitoes kept for assessment of delayed 24 h mortality could not be smeared as most were gravid. Filter paper blood spots were cut using a hole punch (8 mm diameter), placed in an Eppendorf tube with phosphate buffered saline (PBS) solution (600 μl), vortex for 5–10 seconds and incubated at 4°C over night. The following morning an aliquot of the sample (50 μl) was dispensed into each micro plate well, covered and incubated at room temperature for 3 hours. Each well was washed twice with washing buffer (PBS/Tween 0.5%) and filled with blocking buffer (PBS/Casein in NaOH; 200 μl) and incubated for 1 hour. Wells were washed twice with washing buffer and a host-specific conjugate (antihorse IgG H&L; 50 μl) diluted 1:2000 (for antihuman) or 1:250 (for bovine) was added (Kirkegaard and Perry Laboratories). After 1 hour, wells were emptied and washed three times with washing buffer, and ABTS peroxidase substrate (100 μl; Kirkegaard and Perry Laboratories) was added to each well. 30 min after addition of the substrate absorbance was read at 405 nm in ELISA reader (BioTek® Instruments, USA). Each sample was done in duplicate resulting in a maximum of 16 samples per plate, 2 positive and 4 negative controls. Samples were considered positive if absorbance values exceeded the mean plus three standard deviations of the four negative controls (unfed mosquitoes). A visual score based on colour was done and compared with optical density results.

### Statistical analysis

Knockdown and mortality from the laboratory cone and ball tests were expressed as percentages and 95% confidence intervals calculated. For the laboratory repellency tests, protective efficacy (PE) was defined as percentage reduction in the proportion of mosquitoes biting (LSHTM) or landing (Tanzania) in the treatment compared with the control. It was calculated as PE = (1-t/c)*100; where t - response in the treatment, c - response in the control.

For the experimental hut trial, data were entered into an Excel database and transferred to Stata 10 (Stata Corp LP, College Station, TX, USA) for processing and analysis. Logistic regression for proportional data was used to estimate the outcomes, comparing results for treated and untreated blankets, clustering by day, and adjusting for variation between individual sleepers, experimental huts, and blanket body cover score. Estimated proportions were corrected for control mortality using Abbot’s correction. Insecticide induced exophily and blood feeding inhibition in unwashed and 5 times washed treatments were calculated using the respective (unwashed and 5 times washed) controls.

### Ethical approval

The study was approved by London School of Hygiene and Tropical Medicine and the National Ethics Committee of Tanzania (NIMR/HQ/R.8c/Vol. I/24). Written consent was obtained from volunteers participating in the study. During the trial all volunteers were monitored each day for signs of fever or possible side-effects of the ITNs/LLINs.

## Results

### Laboratory study

#### Contact bioassays

**Figure 2:**
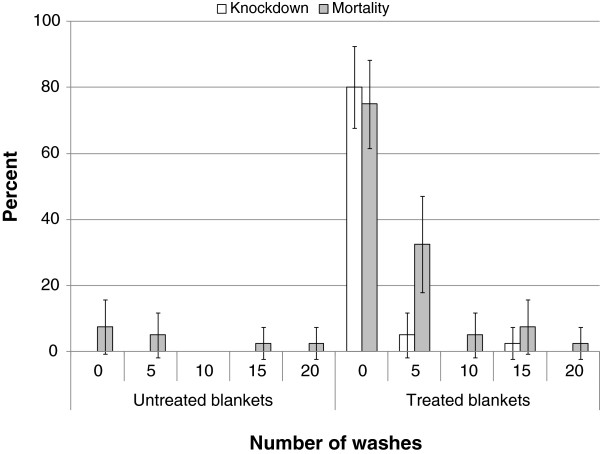
**Arithmetic means of 10 minute knockdown and 24 hour mortality (with 95%Confidence Intervals).** Mean expressed as percentages for materials with 0, 5, 10, 15 or 20 washes tested in WHO cone bioassays.

In cone bioassays carried out at LSHTM (UK) unwashed LLIBs gave 80% knockdown, which decreased to low levels (<10%) after 5, 10, 15 and 20 washes, that were not significantly different from the untreated control. Mortality of all untreated control treatments (unwashed and washed five times) was <10% (p > 0.05; Figure 2). Unwashed LLIBs killed 75% of *An. gambiae* Kisumu. Mortality caused by the LLIB dropped to approximately 30% after five washes and upon further washing was then reduced further to levels comparable to the controls (Figure 2).

**Figure 3:**
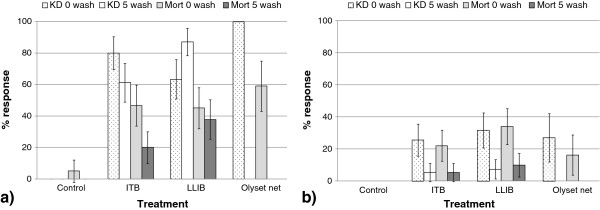
**Knock-down and mortality of *An. gambiae s.s. *Kisumu and *An. arabiensis *Moshi.** ITB = Dipped permethrin blanket 500 mg/m^2^. LLIB = Long-lasting factory treated blanket. KD = Knock-down, Mort = mortality of *An. gambiae s.s.* Kisumu **(a)** and *An. arabiensis* Moshi **(b)**, observed after a 3 minute exposure in ball bioassays to insecticidal blankets washed 0 and 5 times. Olyset nets were not washed.

At KCMUCo (Tanzania) ball bioassays of *An. gambiae* Kisumu showed that the unwashed Olyset net produced significantly greater knockdown (100% knockdown) than the unwashed LLIB (63%) and unwashed ITB (80%) (p < 0.01, Figure 3a). After 5 washes knock-down caused by the LLIB increased significantly from 63% to 87% (p = 0.003), while that of ITB decreased significantly to 61%, (p = 0.002) (Figure 3a). The Olyset net gave the greatest mortality (59%). Mortality observed in response to the unwashed ITB and LLIB were similar at 47% and 45% (p > 0.855, Figure 3a) but decreased after 5 washes to 20% (ITB, p = 0.002) and 38% (LLIB, p = 0.417, Figure 3a).

For *An. arabiensis*, the knockdown in response to all treatments was similar. The Olyset net gave 27% knockdown and this was not significantly different to the unwashed ITB (26%) and LLIB (32%, p > 0.05, Figure 3b). However, knockdown for the LLIB and ITB decreased significantly after 5 washes to 7% and 5% respectively (p < 0.02, Figure 3b). There was no significant difference in mortality caused by the Olyset net, LLIB and ITB (17%, 34% and 22% respectively; p > 0.05; Figure 3b). Although there was a decrease in mortality following 5 washes of the LLIB and ITB, this was not significantly different from the Olyset net (p > 0.05).

#### Arm-in -cage test for repellence

Arm-in-cage tests at LSHTM (UK) showed protection against biting was 100% for the LLIBs that had been subjected to 0, 5, 10, 15 and 20 washes. However, it should be noted that biting on the control (untreated blanket) was low suggesting that protection may be partially due to the thickness of the material itself (Table 2). Unwashed permethrin-treated blankets also provided significant protection against mosquito landings in comparison to untreated blankets (79%; p < 0.01). The level of protection against landings stayed high after 5 washes (75%), but declined to 41% after 10 washes and 33% after 20 washes.

**Table 2:** **Number of *An. gambiae *** **landing and mosquito bites received through insecticide treated material**

**Number of washes**	**Landing**			**Biting**			**Permethrin (mg/m**^**2**^**)**	**Permethrin reduction (%)**
	**Control**	**Treatment**	**PE**	**Control**	**Treatment**	**PE**		
0	38	8	78.95	7	0	100.00	2154.0	-
5	40	10	75.00	8	0	100.00	1248.9	42.0
10	34	20	41.18	6	0	100.00	728.0	66.2
15	36	22	38.89	7	0	100.00	639.8	70.3
20	40	27	32.50	5	0	100.00	563.4	73.8

PE = protective efficacy, after 0, 5, 10, 15 and 20 washes along with the quantity of permethrin in the material determined by HPLC analysis.

**Figure 4:**
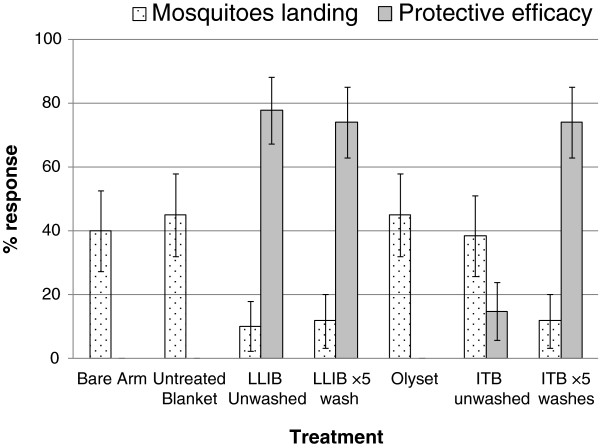
**Landing and protective efficacy of unwashed and washed blankets and Olyset LLIN for *An. gambiae.*** 5 minutes arm-in-cage bioassay, ITB = Dipped permethrin blanket 500 mg/m^2^. LLIB = Long-lasting factory treated blanket.

Arm-in-cage tests were also carried out in Tanzania on blankets washed 0 and 5 times as well as Olyset nets. Fewer mosquitoes landed on arms with unwashed LLIB (10%) compared to untreated blanket (45%) and bare arm (40%, p < 0.001, Figure 4). The protective efficacy of LLIB either unwashed (78%) or washed 5 times (74%) was superior to the Olyset LLIN (0%). There was no difference in the number of mosquitoes landing on arms wrapped in Olyset net (45%) compared to those landing on arms wrapped in untreated blanket (45%) or bare arm (40%, p > 0.05). The ITB showed little protective efficacy when unwashed (15%) but was more repellent after 5 washes, reducing mosquito landing from 38% to 12% (p = 0.001).

### HPLC analysis

The unwashed LLIBs contained high levels of permethrin (2154 mg/m^2^); however, after 5 washes this decreased by 42% to 1248.9 mg/m^2^, and continued to decrease with subsequent washes by 66% after 10 washes, and by 74% after 20 washes (Table 2). This decline in active ingredient content correlated with reduced performance in bioassays.

### Experimental hut trial

A total of 2016 mosquitoes were collected over 42 nights; of the total catch *An. arabiensis* mosquitoes (78.52%) were most abundant accounting over 3 times of *Cx. quinquefasciatus* (21.48%) collected.

#### Anopheles arabiensis

The overall mortality of *An. arabiensis* collected in huts with all types of permethrin blanket was scored at 22-29%, and was significantly greater than the mortality associated with untreated blankets of 1-2% (Table 3). Taking into account hut, volunteer, blanket coverage and collection night, the mortality in huts with Olyset nets (31%) was not significantly greater than unwashed LLIBs (26%, p = 0.501), unwashed ITBs (29%, p = 0.265) or washed ITBs (27%, p = 0.062). However, after washing, LLIBs did have a significantly lower mortality rate (22%, p = 0.037) compared to the Olyset net (Table 3).

**Table 3:** **Results obtained for *Anopheles arabiensis *** **(n = 42 nights) in the experimental huts in Moshi**

**Hut treatment**	**Unwashed control**	**Washed control**	**Unwashed LLIB**	**Washed LLIB**	**Unwashed ITB**	**Washed ITB**	**Olyset net**
Initial dose of permethrin	0 mg/m^2^	0 mg/m^2^	2000 mg/m^2^	2000 mg/m^2^	500 mg/m^2^	500 mg/m^2^	1000 mg/m^2^
Number of washes	0	5	0	5	0	5	0
% volunteer body cover (95% CI)	81 (77–85)	83 (80–87)	79 (74–83)	78 (73–82)	79 (72–85)	79 (72–85)	100
**Total females caught**	**192**	**205**	**224**	**237**	**240**	**197**	**288**
Females caught/night	5	5	5	6	6	5	7
**Total females in verandah and exit traps**	**155**	**170**	**194**	**210**	**203**	**169**	**258**
Exophily (%)	81^ad^	83^d^	87^abd^	89^abc^	85^acd^	86^acd^	90^c^
95% Confidence limits	73-86	70-91	80-91	82-93	79-89	79-91	83-94
**Total females blood fed**	**67**	**85**	**56**	**50**	**48**	**54**	**51**
Blood fed (%)	35^ab^	41^a^	25^bc^	21^cd^	20^cd^	27^bc^	18^d^
95% Confidence limits	26-45	33-50	19-32	17-26	14-28	16-42	13-24
Blood feeding inhibition (%)	-	-	29	49	43	34	49
**Total females dead**	**4**	**3**	**59**	**53**	**70**	**53**	**90**
Overall mortality (%)	2^a^	1^a^	26^bc^	22^b^	29^bc^	27^bc^	31^c^
95% Confidence limits	1-5	0-5	19-36	15-31	21-39	21-34	24-39
Corrected for control (%)	-	0	24	21	27	25	29
Unfed mortality	3 (1–8)	3 (1–8)	30 (20–41)	23 (16–32)	31 (21–43)	30 (23–38)	32 (24–43)
Blood fed mortality	0	0	16 (7–32)	20 (9–39)	21 (11–36)	19 (12–28)	25 (14–42)

Numbers in the same row sharing a letter superscript do not differ significantly (*P* > 0.05).

Blood-feeding inhibition was greatest in huts containing LLIBs washed 5 times and Olyset net, corresponding to 49% inhibition compared to washed control blankets. There was no significant difference between the LLIB washed 5 times (49% inhibition) or the ITB washed 5 times (34%) p = 0.080.

In all huts, over 80% of all *An. arabiensis* mosquitoes were collected from the screened veranda and exit traps indicating a high level of exophily regardless of the presence of permethrin inside the huts (Table 3). However, there was a significantly greater level of exophily in the huts containing Olyset nets than the untreated controls (Olyset: 90%; unwashed control: 81%, p = 0.002; washed control 83%, p = 0.001). There was no significant difference between the treated blankets compared with untreated controls (Table 3).

#### Culex quinquefasciatus

Mortality rates of *Cx. quinquefasciatus* in all treatment huts were only 8-12%, lower than the mortality rate recorded with *An. arabiensis*. However, mortality rates associated with all permethrin treated blankets were significantly greater than those with unwashed controls (Table 4). Mortality rates in huts with treated blankets were not significantly different to those in huts with Olyset nets (Table 4).

**Table 4:** **Results obtained for *Culex quinquefasciatus *** **(n = 42 nights) in the experimental huts in Moshi**

**Hut Treatment**	**Unwashed control**	**Washed control**	**Unwashed LLIB**	**Washed LLIB**	**Unwashed ITB**	**Washed ITB**	**Olyset net**
Initial dose of permethrin	0 mg/m^2^		2000 mg/m^2^	2000 mg/m^2^	500 mg/m^2^	500 mg/m^2^	1000 mg/m^2^
Number of washes	0	5	0	5	0	5	0
% volunteer body cover (95% CI)	81 (77–85)	83 (80–87)	79 (74–83)	78 (73–82)	79 (72–85)	79 (72–85)	100
**Total females caught**	**62**	**79**	**59**	**53**	**60**	**58**	**62**
Females caught/night	2	2	1	1	1	1	2
**Total females in verandah and exit traps**	**25**	**27**	**34**	**25**	**31**	**26**	**50**
Exophily (%)	40^ac^	34^a^	58^c^	47^ac^	52^ac^	45^ac^	81^b^
95% Confidence limits	25-57	22-49	42-72	36-59	35-68	28-63	66-90
**Total females blood fed**	**45**	**67**	**36**	**27**	**29**	**22**	**7**
Blood fed (%)	73^a^	85^b^	61^a^	51^ad^	48^ad^	38^cd^	11^e^
95% Confidence limits	57-84	74-91	43-76	36-65	34-63	25-53	5-25
Blood feeding inhibition (%)	-	-	16	40	34	55	85
**Total females dead**	**0**	**1**	**6**	**5**	**5**	**7**	**4**
Overall mortality (%)	0^a^	1^b^	10^bcd^	9^bcd^	8^d^	12^cd^	6^bd^
95% Confidence limits	-	0-10	4-24	3-25	3-21	6-23	2-17
Corrected for control (%)	-	1	10	9	8	12	6
Unfed mortality	0	0	8 (1–53)	26 (9–54)	15 (4–43)	16 (6–37)	19 (10–34)
Blood fed mortality	0	0	0	4 (0–25)	0	0	0

Numbers in the same row sharing a letter superscript do not differ significantly (*P* > 0.05).

Overall, blood-feeding rates across all treatments were greater for *Cx. quinquefasciatus* (53.8%) than for *An. arabiensis* (26.0%). Olyset nets reduced blood feeding by 85% compared with untreated controls, which was significantly more effective than all blanket treatments. Feeding inhibition of *Cx. quinquefasciatus* was high for LLIBs washed 5 times (40%, p = 0.001) and ITBs washed 5 times (55%, p = 0.001) compared to washed untreated blankets (85% blood-fed). Washing did not affect blood feeding inhibition. *Culex quinquefasciatus* exit rates were 45-58% in huts containing treated blankets, which was lower than the >85% exit rates seen in *An. arabiensis.* Only huts containing Olyset nets had a significantly greater exit rate compared with the controls and treated blankets (Table 4).

#### Blanket usage

There was no difference on how volunteers covered their bodies using the different blanket treatments (p > 0.05, Tables 3 and 4). Generally the mean body coverage was high at >80% for 6 of 7 volunteer sleepers. There was nightly variation in blanket body coverage with all sleepers having a maximum score of 100% (i.e. total body coverage) on some nights and a minimum cover of 0 for one sleeper (i.e. on one night the blanket did not cover any parts of the body).

#### Blood meal source identification

A total of 61 mosquitoes, 48 of which were *An. arabiensis* and 13 *Cx. quinquefasciatus*, were analyzed for blood meal sources. 32% of *An. arabiensis* and 62% of *Cx. quinquefasciatus* contained blood from humans compared with 67% and 31% fed on cattle respectively (Table 5). The percentage sum is greater than 100% because two (4%) *An. arabiensis* and two (15%) *Cx. quinquefasciatus* mosquitoes contained blood from both hosts (Table 5). The number of *An. arabiensis* analysed for blood meal hosts were too few to assess whether there was any difference in human or cow fed between treatments.

**Table 5:** Blood meal host sources of mosquitoes collected from experimental huts

**Treatment**	***An. arabiensis***				***Cx. quinquefasciatus***			
	**Cow fed**	**Human fed**	**Mixed feeding**	**Goat fed**	**Cow fed**	**Human fed**	**Mixed feeding**	**Goat fed**
C-UN	5 (50%)	5 (50%)	0	0	0	3 (100%)		0
C	6 (75%)	2 (25%)	0	0	0	3 (100%)		0
LLIB-UN	6 (55%)	4 (36%)	0	1 (9%)	0	0		0
LLIB-W	8 (100%)	0	0	0	0	0		0
ITB-UN	3 (60%)	1 (20%)	1 (20%)	0	0	1 (100%)		0
ITB-W	1 (100%)	0	0	0	3 (37.5%)	3 (37.5%)	2 (25%)	0
Olyset net	3 (37%)	4 (50%)	1 (13%)	0	1 (8%)	0		0

## Discussion

In experimental huts mortality levels may seem disappointingly low for all treated blankets (LLIB and ITB) with mortality no higher than 29% with any treatment in experimental huts. However, the positive control Olyset LLIN only killed 31%, which was not significantly greater than the unwashed LLIB. *Anopheles arabiensis* from the study site were partially resistant to pyrethroids due to elevated metabolic mechanisms (Matowo unpolished data); [31], which may partly explain the low mortality rates. In areas of pyrethroid susceptible *An. gambiae* we predict much higher levels of mortality for the blankets, as has been observed elsewhere for Olyset [32]. Moreover, *An. arabiensis* at the same site was shown to be killed to a lesser extent than *An. gambiae s.s.* by pyrethroid ITNs from 2005–2007, before resistance had developed. This low mortality was attributed to behavioural avoidance of treated materials through reduced persistence in feeding on humans partially protected by ITNs [27]. A recent unpublished IRS study found that of 165 *An. arabiensis* captured over 2 months, only 34% (22–49) were blood-fed in the unsprayed hut, where the sleeper had no mosquito net. This may also indicate that a large proportion of *An. arabiensis* entered experimental huts for resting rather than host-seeking.

Washing of blankets did not decrease mortality caused by LLIB or ITB in experimental huts. This may be due to the large dosages of permethrin used to treat the blankets and relatively small number of washes. There was no evidence that the long lasting insecticidal treatment improved wash resistance when compared to the conventional ITB, possibly due to the fewer number of washes done for this study. The LLIB may show superior wash-fastness over the ITB after a greater number of washes. In communities blankets are likely to be washed more frequently than mosquito nets. Bioassay and hut testing at KCMUCo, Tanzania was done with blankets washed five times based on the large decrease in efficacy observed in bioassays done at LSHTM. Future studies should determine efficacy after a greater number of washes, preferably comparing LLIB with ITB of an equivalent dosage. Some formulation work may be required to ensure the insecticide is retained in the material over greater than five washes. The number of *Culex quinquefasciatus* that entered the experimental huts and the mortality rates were low for all insecticide treatments.

The ball bioassay of unwashed LLIBs and ITBs produced similar levels of mortality to the Olyset LLIN (<80%). Although the ball test ensures contact with netting for resting mosquitoes for the entire duration, a longer exposure time than 3 minutes used here may be required to achieve >80% mortality. The finding that performance was equivalent to Olyset means that under-dosing of blankets was unlikely.

Arm-in-cage bioassays were done to determine the spatial repellent activity and therefore protective efficacy of treated blankets against host-seeking mosquitoes. LLIB was highly repellent, and even after 5 washes outperformed the unwashed ITB. Surprisingly, Olyset LLIN, when wrapped on an arm, provided no protective efficacy against landing of pyrethroid susceptible *An. gambiae* Kisumu. Lack of protection against landing and biting mosquitoes when LLIN contacts the skin is known; a complete lack of biting protection of deltamethrin and permethrin LLINs was reported by Faulde *et al*. [33] against *Aedes aegypti*. This inability of LLINs in contact with the body to prevent landing and biting mosquitoes suggests low levels of spatial repellence from permethrin. This observation highlights the need for a combination of insecticides and repellents to enhance the protective efficacy of treated materials such as blankets and including bed nets [34-36]. In experimental huts the LLIB that had been washed 5 times successfully reduced the level of blood-feeding by 49% compared with the untreated blanket and was equivalent to the Olyset LLIN against *An. arabiensis* in terms of protection. The lack of protection against landing mosquitoes in laboratory studies suggests the Olyset provided personal protection in huts through irritancy after mosquito contact with the net and had limited spatial repellency. The excito-repellent effect of Olyset was sufficient to induce greater exiting compared to the untreated controls. Greater induced exiting in huts with the Olyset net, than treated blankets, was most likely due to the larger surface area of the mosquito net (relative to the size of a room). The 5 times washed LLIB also provided significant protection against *Cx. quinquefasciatus* compared to untreated blankets but was not as good as the Olyset net in terms of personal protection for the sleeper. Elsewhere, several studies have documented a reduction in mosquito blood feeding through the use of impregnated bedding. Rowland *et al.*[10] reported 70% reduction in *An. nigerrimus* feeding success by individuals sleeping under permethrin treated chaddars. Similarly, the use of deltamethrin impregnated bed sheets reduced blood feeding in *An. nigerrimus, An. stephensi, An. pulcherrimus* by over 20% [5].

On average, almost 80% of the body of each volunteer was covered during the trial providing both a physical and chemical barrier against host seeking mosquitoes. The trial was carried out from July-September, which is the cool season in Northern Tanzania. Although indoor temperature was not recorded in this study, data from 2010 showed mean nightly temperature (19:00 – 06:30 Hrs.) of 23.9°C indoors, 20.7°C outdoors in July; 24.4°C indoors, 20.8°C outdoors in August. This is relatively cool for sub-Saharan Africa and is probably why body coverage was so high. Treated blankets may be less well used and therefore less effective in warmer climates and the thickness may need to be adjusted according to region. Additionally, this lower temperature may have meant that some *An. arabiensis* may have entered huts for shelter during this time rather than host-seeking as mean temperature was approximately 3°C warmer inside. Manguin [37] reports a preference for indoor resting by mosquito species in low temperatures and this may have affected the results in our study.

The finding that a greater proportion of *An. arabiensis* were cattle-fed than human-fed confirms the zoophilic tendencies of the species previously observed in the study area [38]. Feeding in *An. arabiensis* is known to be highly flexible with blood-feeding recorded on human beings, cattle, goats and other animals [38-42]. Preference for a host is most likely related to host availability. The high proportion of cattle-fed *An. arabiensis* indicates that mosquitoes were feeding elsewhere and then entering huts for resting, possibly due to warmer temperatures indoors. Despite the majority of blood-feeding being on cattle, the relative blood-feeding inhibition estimates should be relevant provided that an equal number of cattle-fed *An. arabiensis* entered each hut. This is likely to mean that overall blood-feeding inhibition is underestimated as a treatment could prevent blood-feeding on the sleeper, i.e. 100% protection, but the proportion that entered the hut after feeding on cattle would be included, therefore, reducing the perceived level of protection. It is possible that a proportion of the human-fed *An. arabiensis* also fed elsewhere and entered the huts, but this is considered less likely as the nearest cattle shed is 50 m away from the huts, compared with 300 m to the nearest human house. Blood meal analysis, therefore, should be considered as an essential component of experimental hut trials when determining the protective efficacy of indoor interventions, ideally with blood-meals identified to the individual level through use of DNA fingerprinting or similar method [43]. It would have been preferable to analyse the blood-meal source for all mosquitoes collected in huts, but this was not possible as delayed mortality was recorded 24 h after collection, by which time most were gravid.

## Conclusions

When used to cover a large proportion of the body, insecticide treated blankets have a significant impact in terms of vector mortality and personal protection for the sleeper against malaria and filariasis vectors. Countries in sub-Saharan Africa have observed a rapid scale-up of mosquito bed nets with recent campaigns aiming at covering over 80% of the risk populations. Although this observation has led to a significant increase in numbers of nets owned per household, net usage has not mirrored such an increase with factors like temperature, humidity and season reported to affect usage. This low usage may not be enough to achieve a mass protection effect of treated bed nets in different endemic settings. Long-lasting insecticidal blankets and bed sheets are useful in such settings to overcome the limitation of bed net usage as the majority are likely to use blankets/sheets at night against the cool night temperatures. Moreover long-lasting insecticide treated blankets may be particularly useful where use of LLINs is not suitable, such as refugee camps or nomadic populations. Pyrethroid resistance in *An. gambiae* is widespread across much of Africa. This study demonstrated that pyrethroid blankets can provide considerable personal protection even in areas of partial pyrethroid resistance. Nevertheless, alternative repellent insecticides that are safe for humans should be investigated for use on blankets.

## Competing interests

All authors declare that they have no competing interests.

## Authors’ contributions

JK and RO supervised the study and participated in insect collections and bioassays, analysed the data and drafted the manuscript. Additionally JK performed blood meal ELISA of collected mosquitoes. HK carried out the HPLC analysis and interpretation and provided comments on the manuscript. AK and VC supervised bioassays, assisted in data analysis and provided comments on manuscript versions. EI contributed to overall study design and provided inputs on manuscript. JM, SM and FM provided critical inputs on the different versions of the manuscript. MR and JL helped to design the study, provided major inputs into the data analysis and provided critical inputs on the different versions of the manuscript. All the authors read and approved the final manuscript.
